# Salivary Metabolomics for Systemic Cancer Diagnosis: A Systematic Review

**DOI:** 10.3390/metabo13010028

**Published:** 2022-12-23

**Authors:** Kacper Nijakowski, Jakub Zdrojewski, Monika Nowak, Dawid Gruszczyński, Filip Knoll, Anna Surdacka

**Affiliations:** 1 Department of Conservative Dentistry and Endodontics, Poznan University of Medical Sciences, 60-812 Poznan, Poland; 2 Student’s Scientific Group in Department of Conservative Dentistry and Endodontics, Poznan University of Medical Sciences, 60-812 Poznan, Poland

**Keywords:** saliva, metabolomics, metabolome, metabolites, cancer, carcinoma, neoplasm, tumour, biomarkers, oncological diagnostics

## Abstract

Cancers are the leading cause of death worldwide. The most common cancers include breast, lung, and colorectum. Salivary metabolome profiling is a novel non-invasive method in oncological diagnosis. This systematic review was designed to answer the question “Are salivary metabolites reliable for the diagnosis of systemic cancers?”. Following the inclusion and exclusion criteria, nineteen studies were included (according to PRISMA statement guidelines). Changes in salivary metabolome were most commonly determined in patients with breast cancer, gastrointestinal cancers, and lung cancer. Most studies involved unstimulated whole saliva as the diagnostic material, evaluated by different spectroscopic methods. Among the found saliva metabolites, the alterations in the metabolic pathways of amino acids and polyamines were most frequently observed, which showed significant predictive values in oncological diagnostics. The most frequently encountered risks of bias were the absence of data regarding blinding, sample size justification, and randomisation. In conclusion, salivary metabolites seem to be potentially reliable for detecting the most common systemic cancers. However, further research is desirable to confirm these outcomes and to detect new potential metabolic biomarkers in saliva.

## 1. Introduction

Cancers are classified into a group of diseases in which the cells divide uncontrollably, and the newly formed cells do not differentiate into the normal cells characteristic of particular tissues [1]. Cancer cells are able to spread through the blood and lymphatic vessels [2]. Most cancers are caused by the mutations that occur over time due to ageing or environmental exposures. The process of cell tumorigenesis is the result of the interaction between individual genetic factors and external factors, which can be divided into the following groups:

− Physical agents (physical carcinogens, e.g., ultraviolet and ionising radiation)
− Chemical agents (chemical carcinogens, e.g., asbestos, components of tobacco smoke, alcohol, aflatoxin as food contamination, and arsenic as drinking water contamination)
− Biological agents (biological carcinogens, e.g., infections caused by certain viruses, bacteria or parasites) [3,4,5].

In 2020, cancers were the leading cause of death worldwide [6]. The most common cancers involved breast (11.7% of cases), lung (11.4%), and colorectum (10.0%). Lung cancer has the highest mortality rate among all cancers (18%), followed by colorectal cancer (9.4%) and liver cancer (8.3%) [7].

In the case of cancers, early diagnosis is crucial, as it would allow the treatment implementation at the localised stages of the disease [8]. The gold diagnostic standard for most tumours is biopsy with histopathological evaluation of the collected samples [9]. However, this examination might have some disadvantages, including invasiveness, the need to use special equipment, and errors in sample collection and evaluation due to the heterogeneous structure of cancer tissue. Therefore, it is desirable to develop a modern screening method that would allow early and minimally invasive detection of cancerous lesions [10,11].

In recent years, non-invasive studies, i.e., saliva or exhaled breath testing, have revealed progress in discovering biomarkers for various oncological diseases, such as breast cancer, lung cancer, gastrointestinal cancer, and oral cancer [12,13,14]. Saliva is a biofluid that performs many functions, e.g., pre-digesting food, moisturising the oral cavity, and protecting it from microorganisms. It is secreted by the parotid, sublingual and submandibular salivary glands, and minor salivary glands [15]. During the saliva collection, patients are not accompanied by anxiety and discomfort. In addition, professional personnel are not required, and saliva samples are easy to store [16,17]. Saliva is widely used for disease diagnosis due to its more favourable stability than blood serum [18]. It contains molecules that can be potentially associated with the disease course and facilitate diagnosis and prognosis, including proteins, mRNA, miRNA, enzymes, hormones, antibodies, antimicrobial constituents, growth factors, and metabolites [15,19,20,21].

Metabolomics, measuring intracellular metabolites, helps to determine cellular function [22]. The metabolites can be determined using nuclear magnetic resonance (NMR) spectroscopy and mass spectrometry (MS) combined with gas chromatography (GC), capillary electrophoresis (CE), or high-performance liquid chromatography (HPLC) [23,24]. The metabolic markers from different biochemical pathways can be used for screening and differentiation in oncological diagnosis [23]. Salivary metabolome profiling is a novel non-invasive method. The previous systemic review suggested that the salivary biomarkers of the impaired metabolic pathways (such as amino acid metabolism, polyamine metabolism, choline metabolism) can be used reliably for the early diagnosis and monitoring of patients with oral squamous cell carcinoma [25].

Amino acid metabolism has a significant effect on cancer cells. The primary role of amino acids is to provide substrates for the biosynthesis of proteins and nucleic acids and to participate in the metabolism of carbohydrates and lipids. They also take part in non-enzymatic antioxidant mechanisms (via glutathione synthesis) and epigenetic modifications (mainly involving S-adenosylmethionine as a methyl group donor) [26,27,28]. In turn, polyamines interacting with various negatively charged macromolecules stimulate the biosynthesis of nucleic acids and proteins in cells. By doing so, they affect the growth, proliferation, and differentiation of cells, including cancer cells. In addition, they are components of cytoplasmic membranes, favourably affect the transport of metabolites through them, and counteract their degradation. They can also act as “scavengers” of free radicals [29,30,31]. Moreover, metabolites associated with impaired choline metabolism are thought to have a significant impact on reprogramming the metabolism of cancer cells and disrupting signal transmission between them. This can result in faster tumour progression and malignancy [32].

The present systematic review was designed in order to answer the question “Are salivary metabolites reliable for the diagnosis of systemic cancers?”.

## 2. Materials and Methods

### 2.1. Search Strategy and Data Extraction

A systematic review was conducted up to 25 October 2022, according to the Preferred Reporting Items for Systematic Reviews and Meta-Analyses (PRISMA) statement guidelines [33], using the databases PubMed, Scopus and Web of Science. The search formulas included:

− For PubMed: (cancer OR carcinoma OR neoplasm OR tumour OR tumor OR oncology) AND saliva AND (metabolite OR metabolomics)
− For Scopus: TITLE-ABS-KEY((cancer OR carcinoma OR neoplasm OR tumour OR tumor OR oncology) AND saliva AND (metabolite OR metabolomics))
− For Web of Science: TS=((cancer OR carcinoma OR neoplasm OR tumour OR tumor OR oncology) AND saliva AND (metabolite OR metabolomics)).

Records were screened by the title, abstract, and full text by two independent investigators. Studies included in this review matched all the predefined criteria according to PICOS (“Population”, “Intervention”, “Comparison”, “Outcomes”, and “Study design”), as shown in Table 1. A detailed search flowchart is presented in Section 3. The study protocol was registered in the international prospective register of systematic reviews PROSPERO (CRD42022370448).

### 2.2. Quality Assessment and Critical Appraisal for the Systematic Review of Included Studies

**Figure 1:**
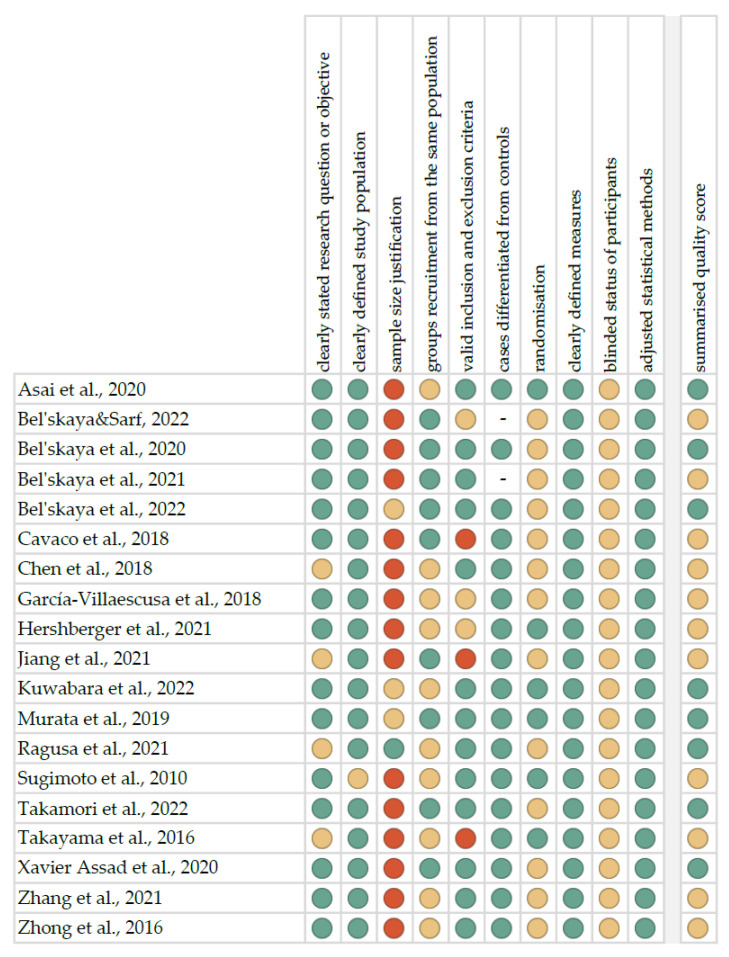
Quality assessment, including the main potential risk of bias (risk level: green—low, yellow—unspecified, red—high; quality score: green—good, yellow—intermediate, red—poor) [36,37,38,39,40,41,42,43,44,45,46,47,48,49,50,51,52,53,54].

The risk of bias in each individual study was assessed according to the “Study Quality Assessment Tool” issued by the National Heart, Lung, and Blood Institute within the National Institute of Health [34]. These questionnaires were answered by two independent investigators, and any disagreements were resolved by discussion between them. The summarised quality assessment for every single study is reported in Figure 1. The most frequently encountered risks of bias were the absence of data regarding blinding (all studies), sample size justification (fifteen studies), and randomisation (thirteen studies). Critical appraisal was summarised by adding up the points for each criterion of potential risk (points: 1—low, 0.5—unspecified, 0—high). Eight studies (42.1%) were classified as having “good” quality (≥80% total score) and eleven (57.9%) as “intermediate” (≥60% total score).

The level of evidence was assessed using the classification of the Oxford Centre for Evidence-Based Medicine levels for diagnosis [35]. All of the included studies have the third or fourth level of evidence (in this five-graded scale).

## 3. Results

**Figure 2:**
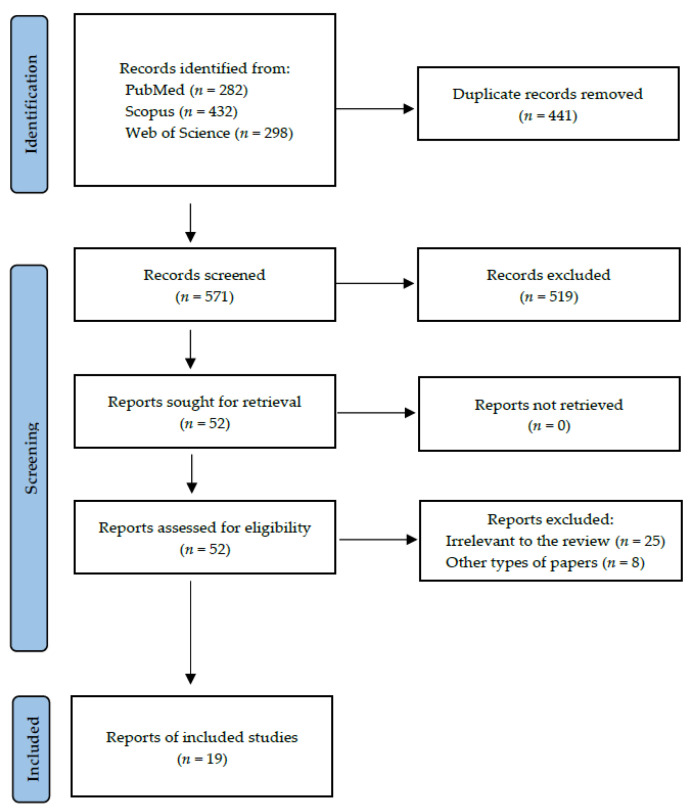
PRISMA flow diagram presenting search strategy.

Following the search criteria, our systematic review included nineteen studies, demonstrating data collected in nine different countries from a total of 2513 participants with diagnosed systemic cancers (including 1528 females and 856 males, and 129 patients without reported gender). Figure 2 shows the detailed selection strategy of the articles. The inclusion and exclusion criteria are presented in Section 2.

From each eligible study included in the present systematic review, we collected data about its general characteristics, such as year of publication and setting, involved participants, oncological diagnosis, inclusion and exclusion criteria, and TNM (tumour-node-metastasis) staging (Table 2). Changes in salivary metabolome were most commonly determined in patients with breast cancer, gastrointestinal cancers, and lung cancer. Table 3 presents the detailed characteristics considering types of saliva, methods of collection, centrifugation, storing, and laboratory analysis, as well as potential salivary metabolites for systemic cancers. Most studies involved unstimulated whole saliva as the diagnostic material, evaluated by different spectroscopic methods. Included studies reported various ways of processing saliva—it was most often centrifuged and stored at −80 °C until analysis. Additionally, predictive parameters for most discriminant metabolites from included studies were reported in Table 4. Among the found saliva metabolites, the alterations in the metabolic pathways of amino acids and polyamines were most frequently observed, which showed significant predictive values in oncological diagnostics.

## 4. Discussion

Our systematic review discusses the most recent studies on the use of saliva metabolome in the diagnosis of systemic cancers, such as breast cancer, gastrointestinal cancers, lung cancer, and others.

### 4.1. Breast Cancer

Breast cancer is the most commonly diagnosed cancer and the leading cause of cancer-related mortality in women worldwide [7]. The diagnostic approach involves self-control, physical examination, and breast imaging, especially ultrasound, mammography, and magnetic resonance. However, breast biopsy with histopathological evaluation remains the only test that can confirm the diagnosis [55,56,57]. Besides, the utilization of highly sensitive and specific metabolomics-based biomarkers could be employed as a new screening tool for patients with breast cancer.

The study by Takayama et al. [42] reported the altered levels of salivary polyamines in patients with breast cancer (BC) before and after their operation compared with the healthy volunteers. The particular polyamines (such as spermine, cadaverine, spermidine, acetylspermine, *N*^1^-acetylspermidine, and *N*^8^-acetylspermidine) strongly correlated with BC patients. Interestingly, *N*^1^-acetylspermidine levels were decreased and *N*^8^-acetylspermidine levels were increased after the surgical treatment. Therefore, the ratio of *N*^8^-acetylspermidine/(*N*^1^-acetylspermidin + *N*^8^-acetylspermidine may be adopted as an index of a health status after surgical operation with both the sensitivity and specificity of nearly 80% based on the ROC analysis (sensitivity 79.1%, 95% CI: 71.5–86.7; specificity 80.0%, 95% CI: 72.3–87.7).

Murata et al. [39] explored the potential salivary metabolites to discriminate patients with invasive carcinoma of the breast (IC), patients with ductal carcinoma in situ (DCIS), and healthy controls, based on multiple logistic regression and the ADTree-based machine learning methods. Among 31 metabolites altered for IC, the top eight ranked metabolites included spermine, *N*^1^-acetylspermine, leucine, glutamine, serine, spermidine, isoleucine, and *N*^1^-acetylspermidine. Only *N*^1^-acetylspermine revealed significant difference also between DCIS and IC. In turn, spermine determined the highest predictive value for IC in comparison to the healthy subjects. Additionally, in the machine learning methods with the higher predictive power, spermine and ribulose-5-phosphate were important discriminant factors in differentiating IC from the controls (AUC = 0.790, 95% CI: 0.699–0.859).

The study by Xavier Assad et al. [43] identified 31 significantly upregulated metabolites in BC patients, including seven oligopeptides and six glycerophospholipids (PG14:2, PA32:1, PS28:0, PS40:6, PI31:1, and PI38:7). In addition, only three peptides and PG14:2 were elevated before but not after effective treatment. Additionally, Zhong et al. [44] screened the potential salivary metabolites for BC diagnosis and staging. Among 18 significantly differed metabolites, lysophosphatidylcholine (18:1), lysophosphatidylcholine (22:6), and monoacylglycerol (0:0/14:0/0:0) were upregulated with the highest predictive power for BC diagnosis. In turn, in the study by Ragusa et al. [40], overexpression of fucose and mannose, as well as underexpression of galactosamine and glucosamine were determined in BC patients.

Interestingly, Cavaco et al. [38] analysed the discrimination potential of the salivary volatile composition for BC in two distinct geographic regions in Portugal (Madeira Island) and India (Pune). For the Portuguese population, 3-methyl-pentanoic acid, 4-methyl-pentanoic acid, phenol, and p-tert-butyl-phenol were statistically relevant to distinguish BC from the healthy controls, and for Indian population, acetic, propanoic, benzoic acids, 1,2-decanediol, 2-decanone, and decanal. These findings suggest that results obtained in a specific cohort may not be generalised to other populations.

Moreover, the study by Bel’skaya et al. [37] determined changes in the salivary metabolic profile in BC patients. In saliva, the total content of α-amino acids significantly increased with a simultaneous significant decrease in the total level of protein, which might indicate a pronounced elevation in protein catabolism. In the early stages of BC, the significantly higher levels of urea, as well as the lowered levels of total protein and uric acid, were observed. In a similar study on patients with primary resectable breast cancer (T_1-3_N_0-1_M_0_), Bel’skaya and Sarf [36] found that salivary levels of diene conjugate below 3.93 c.u. before treatment could be significant risk factor for tumour recurrence (HR = 1.78, 95% CI: 1.02–3.08).

### 4.2. Gastrointestinal Cancers

Gastrointestinal cancers, including oesophageal, gastric, colorectal, liver, and pancreatic tumours, are some of the most frequently diagnosed cancers worldwide. Unfortunately, the late demonstration of disease symptoms is responsible for the diagnostic delay and worse prognostic outcomes [7,58,59]. In particular, pancreatic cancer is characterised by an almost equal number of deaths as cases due to its non-specific symptoms, difficult early diagnosis, rapid progression, short survival time, and poor prognosis [60,61].

Sugimoto et al. [41] assessed the salivary metabolomic profiles in patients with oral cancer, breast cancer, pancreatic cancer, periodontal disease, and healthy controls, using capillary electrophoresis time-of-flight mass spectrometry. The multiple logistic regression model with five potential metabolic markers for pancreatic cancer had the highest prediction determined by ROC analysis for differentiation from the healthy subjects, followed by the fourteen-element model for breast cancer (AUC = 0.993 vs. 0.973).

Asai et al. [45] evaluated the potential ability of salivary polyamines to detect pancreatic cancer (PC), using capillary electrophoresis–mass spectrometry. Three polyamines (spermine, *N*^1^-acetylspermidine, and *N*^1^-acetylspermine) and 2-aminobutanoate showed significant difference between PC patients and others (healthy controls and patients with chronic pancreatitis). Significantly higher concentrations were observed in stages III and IVb. Additionally, the model including alanine, *N*^1^-acetylspermidine, 2-oxobutyrate, and 2-hydroxybutyrate demonstrated high accuracy in discriminating PC patients from the other groups (AUC = 0.887, 95% CI: 0.784–0.944). The lower levels of alanine and the higher levels of the other three metabolites indicated the increased possibility of PC.

In a study not included in the review by Itakura et al. [62] (reported as conference abstract), among metabolites quantified using liquid chromatography–mass spectrometry, 28 metabolites presented significant differences for PC patients, as well as 22 metabolites for patients with early PC stages I/II and C in comparison to the healthy subjects. Among altered metabolites, polyamines, such as *N*^1^-acetylspermidine and *N*^1,8^-acetylspermidine, amino acids, and intermediate glycolysis metabolites, were included. The authors suggest that the salivary polyamines could be used in early and low-invasive detection systems screening for PC.

Moreover, Kuwabara et al. [48] explored and validated salivary biomarkers to distinguish patients with colorectal cancer (CRC) from patients with adenoma (AD) and healthy subjects, using capillary electrophoresis–mass spectrometry and liquid chromatography–mass spectrometry. Among the acetylated polyamines, *N*-acetylputrescine and *N*^1^-acetylspermine showed high potential to discriminate CRC. Based on the pathway analysis, two significant pathways, including alanine, aspartate, and glutamate metabolism, as well as arginine and proline metabolism, had relatively high impact. The top three discriminating metabolites (*N*-acetylputrescine, 4-methyl-2-oxopentanoate, and 5-oxoproline) were used in both models of the alternative decision tree (ADTree)-based machine learning. The model distinguishing CRC from AD and the controls demonstrated higher AUC than the model for CRC + AD vs. healthy subjects (AUC = 0.879, 95% CI: 0.851–0.907 vs. AUC = 0.860, 95% CI: 0.828–0.891). The authors concluded that salivary metabolomics combined with machine learning could present high accuracy and versatility in CRC detection.

The study by Chen et al. [46] identified ten salivary amino acids to distinguish early and advanced gastric cancer patients from healthy controls, using high performance liquid chromatography–mass spectrometry. The highest concentrations of amino acids were observed in patients with early gastric cancer. Based on these finding, the researchers developed surface-enhanced Raman scattering (SERS) sensors, which showed high accuracy to discriminate EGC, AGC, and healthy subjects (specificity > 87.7% and sensitivity > 80%).

In the pilot study, Bel’skaya et al. [47] determined the potential diagnostic capabilities for salivary volatile organic compounds (VOCs) in detecting gastric and colorectal cancer. Levels of acetaldehyde, methanol, and ethanol in saliva were significantly higher in gastric cancer, as well as acetone in colorectal cancer. The content of 1-propanol and 2-propanol was significantly lowered in patients with CRC compared with the healthy controls. The combination of five salivary VOCs (acetaldehyde, acetone, methanol, 2-propanol, and ethanol) allowed the detection of gastric and colorectal cancer with the sensitivity of 80.0% and 92.3%, respectively, whereas the specificity was 100% in both cases.

Hershberger et al. [49] identified promising salivary metabolites that could discriminate patients with hepatocellular carcinoma (HCC) from patients with cirrhosis and healthy subjects. Acetophenone and octadecanol were significantly decreased in patients with cirrhosis and further in patients with HCC compared with healthy individuals. Additionally, lauric acid, 3-hydroxybutyric acid, threonic acid, glycerol-alpha-phosphate, butylamine, and alpha- tocopherol were lowered in patients with HCC in comparison to the controls. The predictive model including four salivary metabolites (octadecanol, acetophenone, 1-monopalmitin, and 1-monostearin) demonstrated the highest sensitivity and specificity (87.9% and 95.5%, respectively) and the lowest misclassification (7.1%) for HCC patients.

### 4.3. Lung Cancer

Lung cancer is the most frequently occurring cancer and the leading cause of cancer-related deaths among men [7,63]. The early diagnosis of lung cancer remains challenging due to the lack of obvious symptoms and limitations of available diagnostic procedures, which are associated with cancer detection in advanced stages [9,64]. Therefore, there is an urgent need to identify reliable biomarkers for early diagnosis of lung cancer. Identifying salivary metabolomic alterations in lung cancer could be a promising approach for non-invasive disease diagnosis.

The preliminary study by Takamori et al. [52] identified salivary metabolites for distinguishing lung cancer (LC) from benign lung lesions (BLL). Among ten different salivary metabolites, only tryptophan concentrations were significantly decreased in LC patients compared with BLL patients. However, the model including four other metabolites (diethanolamine, cytosine, lysine, and tyrosine) showed higher discriminatory ability for patients with LC in comparison to patients with BLL (AUC = 0.729, 95% CI: 0.598–0.861).

In the early lung cancer patients, Jiang et al. [51] verified the significant dysfunction of the metabolic pathways, such as the amino acid metabolism (including arginine and proline metabolism, arginine biosynthesis, valine, leucine and isoleucine biosynthesis) and the nucleotide metabolism (including purine metabolism and aminoacyl-tRNA biosynthesis). Based on the determined 23 altered salivary metabolites, early LC patients could be differentiated from the healthy subjects with a sensitivity of 97.2% and specificity of 92%.

In turn, Bel’skaya et al. [50] compared the salivary metabolome profiles in lung cancer and chronic obstructive pulmonary disease (COPD) of varying severity, depending on the smoking experience. For example, the salivary levels of diene conjugates differed depending on COPD coincidence and its severity. The smoking factor did not have a significant influence on these changes.

Similarly to BC patients, the study by Ragusa et al. [40] found overexpression of fucose and mannose, as well as underexpression of galactosamine and galactose in LC patients.

### 4.4. Other Tumours

The study by Zhang et al. [53] validated the utility of the salivary amino acids in the diagnosis of papillary thyroid carcinoma (PTC), using ultra-high performance liquid chromatography–high resolution mass spectrometry. The salivary levels of 10 amino acids significantly differed between PTC and the healthy volunteers. The combination of alanine, valine, proline, and phenylalanine demonstrated the improved accuracy for early diagnosis of thyroid cancer with a sensitivity of 91.2% and specificity of 85.2%.

Furthermore, García-Villaescusa et al. [54] investigated the potential relationship between glioblastoma and chronic periodontitis, based on salivary metabolome alterations. In patients with glioblastoma, the significantly elevated salivary levels of metabolites, such as leucine, valine, isoleucine, propionate, alanine, acetate, ethanolamine, and sucrose, were found. In turn, the significantly increased salivary concentrations of caproate, isocaproate + butyrate, isovalerate, isopropanol + methanol, 4-aminobutyrate, choline, sucrose, sucrose + glucose + lysine, lactate + proline, lactate, and proline could be used as biomarkers for periodontal disease.

### 4.5. Study Limitations

The review limitations include the heterogeneity of the study designs, clinical and histopathological diagnoses, as well as laboratory methods of salivary metabolome determination. The most common methodological problem was the lack of justification for the sample size. All studies included statistical analyses with strictly defined levels of proper significance. Some of them performed the advanced statistical methods incorporating machine-learning techniques along with validation of the determined models. Unfortunately, not all studies presented the predictive values with confidence intervals for the potentially proposed markers in the oncological diagnosis. Additionally, the exclusion of the studies reported in conference proceedings and other grey literature might affect the results of this systematic review.

The included studies focused on the wide range of metabolic pathways, making it impossible to compare the observed differences between the particular metabolites. However, alterations involving polyamines (i.e., spermine, spermidine, etc.) were most commonly described. These molecules are known to affect the growth, proliferation, and differentiation of cells, including cancer cells.

Moreover, it should be noted that changes in the saliva metabolome may be very dynamic and dependent on various factors (e.g., oral health status, dietary habits, microbiome activity). As mentioned earlier, it is not possible to extrapolate metabolic changes identified in a particular group to other populations. In addition to these individual features, the conditions related to the processing of saliva in the laboratory (such as collection or processing temperature and duration) are not insignificant. There is also a lack of studies comparing changes in metabolomics for different biological fluids. These aspects are the biggest barriers to introducing non-invasive saliva diagnostics into the clinical practice.

## 5. Conclusions

According to our systematic review, salivary metabolites seem to be potentially reliable to detect the most common systemic cancers, with some abovementioned limitations. However, further research is desirable to confirm these outcomes and to detect new potential metabolic biomarkers in saliva.

## Figures and Tables

**Table 1:** Inclusion and exclusion criteria according to the PECOS.

Parameter	Inclusion Criteria	Exclusion Criteria
Population	patientsaged from 0 to 99 years, both genders	
Exposure	systemic cancers	other neoplasms (e.g., oral cancers)
Comparison	not applicable	
Outcomes	salivary metabolites as markers	other salivary components as markers
Study design	case–control, cohort and cross-sectional studies	literature reviews, case reports, expert opinion, letters to the editor, conference reports
	published after 2000	not published in English

**Table 2:** General characteristics of included studies.

Author, Year	Setting	Study Group (F/M); Age	Control Group (F/M); Age	Oncological Diagnosis	Inclusion Criteria	Exclusion Criteria	TNM Stages
Bel’skaya & Sarf, 2022 [36]	Russia	355 (355/0); 30–39: 34 (9.6%), 40–49: 68 (19.2%), 50–59: 117 (33.0%), 60–69: 105 (29.6%), >70: 31 (8.6%)	–	breast cancer	diagnosis of primary resectable BC	NR	pT1–133 (37.5%), pT2–172 (48.5%), pT3–50 (14.0%); pN0–245 (69.0%), pN1–110 (31.0%)
Bel’skaya et al., 2022 [37]	Russia	487 (487/0); 54.5 (47.0–56.0)	298 (298/0); 49.3 (43.8–56.1)	breast cancer	histologically diagnosed with BC; age 30–70 years; absence of any treatment at the time of the study; absence of signs of active infection (including purulent processes); good oral hygiene; absence of untreated dental caries and periodontal disease; absence of clinically significant concomitant diseases other than cancer pathology (in particular, diabetes mellitus, cardiovascular pathologies, etc.)	any prior treatment, including hormone therapy, chemotherapy, molecularly targeted therapy, radiotherapy, surgery; lack of histological verification of the diagnosis	I–119 (24.4%), IIA–123 (25.3%), IIB–88 (18.1%), IIIA–55 (11.3%), IIIB–47 (9.6%), IV–55 (11.3%)
Cavaco et al., 2018 [38]	Portugal, India	Portugal: 36 (36/0); range: 39–73; India: 30 (30/0); range: 25–76	Portugal: 16 (16/0); range: 18–63, India: 24 (24/0); range: 23–65	breast cancer	NR	NR	NR
Murata et al., 2019 [39]	Japan	IC: 101 (101/0); 54 (34–89); DCIS: 23 (23/0); 49 (39–80)	42 (42/0); 51 (23–80)	breast cancer	histologically diagnosed with BC	any prior treatment, including hormone therapy, chemotherapy, molecularly targeted therapy, radiotherapy, surgery, or alternative therapy; Ctrl: absence of history of any cancer	0–23 (DCIS), IC: I–44 (45.4%), II– 46 (47.4%), III–5 (5.1%), IV–2 (2.1%)
Ragusa et al., 2021 [40]	Italy	BC: 38 (38/0); 54.2 ± 13.0; LC: 30 (8/22); 69.8 ± 10.3	34 (18/16); 46.2 ± 10.8	breast cancer, lung cancer	age > 18 years; BMI of about 25–26 kg/m^2^; established clinical diagnosis of either BC or LC (including mesothelioma)	pregnancy; previous history of other malignancies; in the terminal stage (expected less than 4 weeks old); conditions that might have potentially interfer from a metabolic point of view; simultaneous liver cirrhosis, gastric ulcers, diabetes mellitus, periodontitis	NR
Sugimoto et al., 2010 [41]	U.S.A.	BC: 30 (30/0); 57 (29–77); PC: 18 (NR); 67 (11–87)	87 (27/42, 18 missing); 43 (20–75)	breast cancer, pancreatic cancer	diagnosed with primary disease without metastasis	prior chemotherapy, radiotherapy, surgery or alternative therapy, history of prior malignancy, immunodeficiency, autoimmune disorders, hepatitis or HIV infection	NR
Takayama et al., 2016 [42]	Japan	111 (NR); range: 36–90	61 (NR)	breast cancer	NR	NR	0–16 (14.4%), I–50 (45.0%), IIA–32 (28.8%), IIB–10 (9.0%), IIIA–1 (0.9%), unknown–2 (1.8%)
Xavier Assad et al., 2020 [43]	Brazil	23 (23/0); 47.52 ± 9.79	35 (35/0); 42.00 ± 13.83	breast cancer	not pregnant or lactating; no active oral/dental disease; no prior neoplasia, except for non-melanomatous skin cancers, cervical carcinoma in situ, or benign tumors (e.g., adenomas); no impaired renal function, congestive heart failure, or active infection (e.g., hepatitis and HIV); histopathological diagnosis of BC; Ctrl: normal clinical and imaging findings	Ctrl: abnormal imaging or clinical findings; history of cancer treatment	I–2 (8.7%), II–12 (52.2%), III–5 (21.7%), IV–4 (17.4%)
Zhong et al., 2016 [44]	China	30 (30/0); 53 (32–79)	25 (25/0); NR	breast cancer	diagnosis of BC based on clinical and histopathological criteria; Ctrl: no history of malignancy or relevant breast diseases	History of receiving surgical operation and medication, including chemotherapy, radiotherapy, or alternative therapy	I–7 (23.3%), II–14 (46.7%), III–8 (26.7%), IV–1 (3.3%)
Asai et al., 2018 [45]	Japan	PC: 39 (18/21); 66.1 ± 9.86	Ctrl: 26 (13/13); 50.8 ± 16.4; CP: 14 (3/11); 51.1 ± 12.4	pancreatic cancer	histologically diagnosed with PC	prior treatment in the form of chemotherapy, radiotherapy, surgery, or alternative therapy; prior malignancy	III–6 (15.4%), IVA–12 (30.8%), IVB–21 (53.8%)
Chen et al., 2018 [46]	China	EGC: 20 (7/13); 60 ± 8.6; AGC: 84 (34/50); 53 ± 9	116 (49/67); 35.0 ± 10.0	gastric cancer	clinical diagnosis of GC	diagnosis of other malignancies; metabolic diseases (mainly including diabetes)	EGC: stage I and II, defined as that the tumour invasion confined to the mucosa or submucosa; AGC: stage III and IV, defined as that the tumour invading into the muscularis propria or deeper gastric wall
Bel’skaya et al., 2020 [47]	Russia	GC: 11 (3/8); 56.8 ± 5.5; CRC: 18 (7/11); 58.2 ± 3.8	16 (6/10); 57.1 ± 6.4	gastric cancer, colorectal cancer	age 30–70 years; the absence of signs of active infection (including purulent processes); absence of clinically significant concomitant diseases other than cancer pathology (in particular, diabetes, cardiovascular pathologies); good oral hygiene	any treatment at the time of the study, including surgery, chemotherapy or radiation; lack of histological verification of the diagnosis	GC: IIA–4 (36.4%), IIIA–2 (18.2%), IIIB–3 (27.3%), IV–2 (18.2%); CRC: I–2 (11.0%), IIB–3 (16.7%), IIC–5 (27.8%), IIIC–3 (16.7%), IV–5 (27.8%)
Kuwabara et al., 2022 [48]	Japan	training data: CRC: 117 (53/64); 67.42 ± 11.24; validation data: CRC: 118 (52/66); 69.63 ± 12.14	Ctrl: training data: 1159 (841/318); 45.65 ± 10.15; validation data: 1158 (820/338); 45.19 ± 10.10; AD: training data: 25 (4/21); 66.30 ± 11.07; validation data: 25 (5/20); 61.81 ± 10.40	colorectal cancer	histopathological diagnosis of CRC	prior treatment in the form of chemotherapy; chronic metabolic diseases, e.g., diabetes; histopathological diagnosis of all other types of cancer (adenosquamous cell carcinoma, endocrine carcinoma, lymphoma, etc.)	training data: 0–2, I–30, II (N1)–36, II (N2)–25, III–14, IVa–10; validation data: 0–2, I–31, II (N1)–36, II (N2)–25, III–14, IVa–10
Hershberger et al., 2021 [49]	U.S.A.	37 (7/30); 67.3 (44–94)	Crtl: 43 (16/27); 57.6 (36–77); cirrhosis: 30 (18/12); 58 (33–80)	hepatocellular carcinoma	age > 18 years; liver transplantation for HCC or cirrhosis; surgical resection for HCC or liver biopsy with confirmed cirrhosis and/or HCC; Ctrl: patients attending treatment for hernia with no history of liver disease or liver cancer	NR	NR
Bel’skaya et al., 2021 [50]	Russia	LC: 392 (85/244): ADC: 189 (60/129); 61.0 (56.0–65.0), SCC: 135 (7/128); 59.0 (55.0–66.5), NEC: 68 (18/50); 55.0 (52.0–60.0)	-	lung cancer	age 30–75 years; histological verification of the diagnosis	any treatment at the time of inclusion in the study, including surgery, chemotherapy or radiation	ADC: IA–16 (8.5%), IB–52 (27.5%), IIA + B–23 (12.2%), IIIA–25 (13.2%), IIIB–17 (9.0%), IV–56 (29.6%); SCC: IA–3 (2.2%), IB–28 (20.7%), IIA + B–19 (14.1%), IIIA–34 (25.2%), IIIB–24 (17.8%), IV–27 (20.0%); NEC: IA–5 (7.4%), IB–10 (14.7%), IIA + B–6 (8.8%), IIIA–10 (14.7%), IIIB–17 (25.0%), IV–20 (29.4%)
Jiang et al., 2021 [51]	China	ELC: discovery set: 45 (29/16); 57.8 (13.4); validation set: 44 (29/15); 55.3 (10.9); ALC: 11 (4/7); 70.2 (6.9)	discovery set: 25 (15/10); 52.9 (12.3); validation set: 25 (16/9); 57.3 (15.8)	lung cancer	NR	NR	I–89 (ELC), III–1 and IV–10 (ALC)
Takamori et al., 2022 [52]	Japan	42 (14/28); 63 (39–86)	BLL: 21 (6/15); 62 (43–86)	lung cancer	confirmation of clinical or pathological diagnosis; consulted a dental surgeon before lung surgery; underwent PET/CT for LC	history of malignancy; prior treatment in the form of chemotherapy or radiotherapy at the time of pathological and clinical diagnosis	I–31 (73.8%), II–4 (9.5%), III–4 (9.5%), IV–3 (7.2%)
Zhang et al., 2021 [53]	China	61 (44/17); 44 ± 11	61 (42/19); NR	papillary thyroid cancer	newly diagnosed with PTC; no history of malignancy and immunodeficiency disease; normal thyroid gland function	prior treatment in the form of surgery, long-term chemotherapy, radiation, and drug therapy	NR
García-Villaescusa et al., 2018 [54]	Spain	10 (9/1); 54.7 (26–78)	120 (71/49); 51.8 (19–81)	glioblastoma	age ≥ 18 years; diagnosis of glioblastoma; at least eight teeth	Ctrl: antibiotics intake in the past six months; fewer than eight teeth (excluding third molars); pregnancy; presenting cardiovascular diseases, diabetes mellitus, rheumatoid arthritis, chronic obstructive pulmonary disease, pneumonia, chronic kidney disease, metabolic syndrome, obesity and Alzheimer’s disease	NR

U.S.A., the United States of America; F, female; M, male; -, not applicable; NR, not reported; Ctrl, control group; BC, breast cancer; IC, invasive carcinoma of the breast; DCIS, ductal carcinoma in situ; PC, pancreatic cancer; CP, chronic pancreatitis; GC, gastric cancer; EGC, early gastric cancer; AGC, advanced gastric cancer; CRC, colorectal cancer; AD, adenoma; HCC, hepatocellular carcinoma; LC, lung cancer; ADC, adenocarcinoma; SCC, squamous cell carcinoma; NEC, neuroendocrine cancer; ELC, early lung cancer; ALC, advanced lung cancer; BLL, benign lung lesion; PTC, papillary thyroid carcinoma; BMI, body mass index; HIV, human immunodeficiency virus; PET/CT, positron emission tomography/computed tomography.

**Table 3:** Detailed characteristics of included studies considering methods of collection and analysis of saliva.

Author, Year	Oncological Diagnosis	Type of Saliva and Method of Collection	Centrifugation and Storing	Method of Analysis	Potential Discriminant Metabolites in Saliva
Bel’skaya & Sarf, 2022 [36]	breast cancer	unstimulated whole saliva 5 mL collected by spitting into sterile polypropylene tubes; collection of saliva samples was carried out on an empty stomach after rinsing the mouth with water at 8:00–10:00 a.m.	centrifuged at 10,000× *g* for 10 min, biochemical analysis immediately performed without storage and freezing	StatFax 3300 semi-automatic biochemical analyser	prognostic marker: diene conjugates (level above 3.93 c.u.)
Bel’skaya et al., 2022 [37]	breast cancer	unstimulated whole saliva 5 mL collected by spitting into sterile polypropylene tubes; collection of saliva samples was carried out on an empty stomach after rinsing the mouth with water at 8:00–10:00 a.m.	centrifuged at 10,000× *g* for 10 min, biochemical analysis immediately performed without storage and freezing	StatFax 3300 semi-automatic biochemical analyser	up: total content of α-amino acids, urea; down: total protein, uric acid
Cavaco et al., 2018 [38]	breast cancer	unstimulated whole saliva collected in an 8–mL sterilised glass vials after rinsing the mouth with water in the morning	stored at –80 °C in aliquots of 2 mL until analysis	HS-SPME/GC-MS	Portugal: down: 3-methyl-butanoic acid, 4-methyl-pentanoic acid, phenol, acetic acid, propanoic acid, butanoic acid; India: up: acetic acid, propanoic acid, butanoic acid, 3-methyl-butanoic acid, 4-methyl-pentanoic acid down: 1,2-decanediol, pentanoic acid
Murata et al., 2019 [39]	breast cancer	unstimulated saliva 400 μL collected in a 50 cc polypropylene tube (a polypropylene straw 1.1 cm in diameter was used to assist the saliva collection) after rinsing the mouth with water at 9:00–11:00 a.m.	immediately stored at −80 °C until analysis	CE-TOF-MS	among 31 metabolites the top eight ranked included spermine, *N*^1^-acetylspermine, leucine, glutamine, serine, spermidine, isoleucine, and *N*^1^-acetylspermidine
Ragusa et al., 2021 [40]	breast cancer, lung cancer	unstimulated whole saliva 3 mL collected in a sterilised plastic vial, early in the morning, immediately transferred and centrifuged	centrifuged at 1500 rcf for 10 min, the supernatant was aliquoted in sterilised screw cap plastic vials (0.4 mL of saliva sample each) and stored at −80 °C until analysis	HPAEC-PAD	BC: up: fucose, mannose and galactose, down: glucosamine (*p*-value < 0.001); LC: up: fucose and mannose (*p*-value < 0.001), down: galactose (*p*-value < 0.01), and galactosamine (*p*-value < 0.05)
Sugimoto et al., 2010 [41]	breast cancer, pancreatic cancer	unstimulated whole saliva 5 mL for 5–10 min, spitted into 50 mL Falcon tubes, placed in a Styrofoam cup filled with crushed ice	centrifuged at 2600× *g* for 15 min at 4 °C and spun for 20 min in case of incomplete separation, transferred to two fresh tubes and frozen within 30 min	CE-TOF-MS	BC: C_2_H_6_N_2_, C_30_H_62_N_19_O_2_S_3_, taurine, C_8_H_9_N, lysine, glycerophosphocholine and C_7_H_8_O_3_S (*p*-value < 0.001), C_32_H_48_O_13_, C4H_12_N_5_, cadaverine, putrescine, leucine + isoleucine, tyrosine, proline, aspartic acid, glutamic acid and threonine (*p*-value < 0.01), C_30_H_55_N_27_O_3_S, alpha-aminobutyric acid, alanine, piperideine, phenylalanine, ethanolamine, glycine, ornithine, valine, and serine (*p*-value < 0.05); PC: C_2_H_6_N_2_, C_3_H_7_NO_2_, C_4_H_12_N_5_, C_4_H_9_NO_2_, C_30_H_62_N_19_O_2_S_3_, alpha-aminobutyric acid, alanine, putrescine, methylimidazoleacetic acid, trimethylamine, C_5_H_14_N_5_, taurine, C_4_H_9_N, C_6_H_6_N_2_O_2_, leucine + isoleucine, phenyloalanine, tyrosine, lysine, ethanolamine, gamma-aminobutyric acid, aspartic acid, valine, tryptophan, beta-alanine, glutamic acid, threonine, serine, glutamine, hypoxantine, choline and C_5_H_11_NO_2_ (*p*-value < 0.001), cadaverine, histidine, proline, glycine, Pro-Gly-Pro/Pro-Pro-Gly, C_7_H_12_N_2_O_3_, citrulline, carnitine, glycerophosphocholine and C_7_H_8_O_3_S (*p*-value < 0.01), C_30_H_55_N_27_O_3_S, C_18_H_32_N_6_O_6_, piperidine, ornithine, C_17_H_26_N_4_O_5_, and burimamide (*p*-value < 0.05)
Takayama et al., 2016 [42]	breast cancer	unstimulated whole saliva 1 mL collected into a tube	stored < −20 °C until analysis, centrifuged at 3000× *g* for 10 min after thawing	UPLC-ESI-MS/MS	up: spermine, *N*^1^-acetylspermine and *N*^1^-acetylspermidine (*p*-value < 0.0001), *N*^8^-acetylspermidine and *N*^1^-acetylputrescine (*p*-value < 0.005), *N*^1^*N*^8^-diacetylspermidine, *N*^1^*N*^12^-diacetylspermine, and cadaverine (*p*-value < 0.05)
Xavier Assad et al., 2020 [43]	breast cancer	stimulated whole saliva 5–10 mL collected with a cotton swab (Salivette^®^) for 2 min, placed in a plastic container and packaged in a Styrofoam box with recyclable ice packets for less than 4 h before transport and processing	centrifuged at 3000 rpm for 5 min at 8 °C, stored at −80 °C until analysis	LC-Q-TOF-MS	up: 31 metabolites, including 7 oligopeptides and 6 glycerophospholipids (PG 14:2, PA 32:1, PS 28:0, PS 40:6, PI 31:1, and PI 38:7)
Zhong et al., 2016 [44]	breast cancer	unstimulated whole saliva 2 mL collected at 8:30–10:30 a.m.	centrifuged at 13,500 rpm for 20 min and at 4 °C, stored at −40 °C until analysis	HILIC-UPLC-ESI-MS, RP-UPLC-ESI-MS	up: lysophosphatidylcholine (18:1, 22:6), monoacylglycerol (0:0/14:0/0:0), lysophosphatidylethanolamine (18:2/0:0), histidine, and *N*-acetylneuraminic acid (*p*-value < 0.001), lysophosphatidylcholine (16:0), phosphatidylserine (14:1/16:1) phosphatidylcholine (18:1/16:0), phenylalanine, citrulline, phosphatidylethanolamine (22:/20:4), and 4-hydroxyphenylpyruvic acid (*p*-value < 0.05); down: lysophosphatidylcholine (18:2) and phytosphingosine (*p*-value < 0.001), palmitic amide, acetylphenylalanine, and propionylcholine (*p*-value < 0.05)
Asai et al., 2018 [45]	pancreatic cancer	unstimulated whole saliva 400 µL collected in a 50 cc polypropylene tube (a polypropylene straw 1.1 cm in diameter was used to assist the saliva collection) after rinsing the mouth with water at 8:00–11:00 a.m.	immediately stored at −80 °C until analysis	CE-TOF-MS	up: spermine, *N*^1^-acetylspermidine, *N*^1^-acetylspermine, 2-aminobutanoate
Chen et al., 2018 [46]	gastric cancer	unstimulated whole saliva 4 mL collected after cleaning the mouth	centrifuged at 12,000 rpm for 30 min at 4 °C, 2 mL of the supernatant transferred into centrifuge tubes and stored at –70 °C	HPLC-MS, SERS	both EGC and AGC: up: taurine, glutamine, ethanolamine, histidine, alanine, glutamic acid, proline
Bel’skaya et al., 2020 [47]	gastric cancer, colorectal cancer	unstimulated whole saliva 2 mL collected on an empty stomach after rinsing the mouth with water at 8:00–10:00 a.m.	centrifuged at 10,000× *g* for 10 min, biochemical analysis immediately performed without storage and freezing	capillary gas chromatography	GC: up: acetaldehyde, acetone, methanol, ethanol, 1-propanol, 2-propanol and triene conjugates, down: diene conjugates; CRC: up: acetone, ethanol and triene conjugates, down: 1-propanol, 2-propanol, diene conjugates
Kuwabara et al., 2022 [48]	colorectal cancer	unstimulated saliva 400 μL collected and stored in 50 mL polypropylene tubes (a polypropylene straw 1.1 cm in diameter was used to assist the saliva collection) at 9:00–11:00 a.m.	immediately stored at −80 °C until analysis	CE-TOF-MS, LC-QQQ-MS	up: *N*-acetylputrescine, *N*^1^*N*^8^-diacetylspermidine, alanine, 5-oxoproline, *N*^1^-acetylspermine, *N*^8^-acetylspermidine, succinate, 5-hydroxy-4-methylpentanoate and 2-hydroxypentanoate; down: *N*-acetylneuraminate, hexanoate, urate, dihydroxyacetone phosphate, aspartate, and beta-alanine
Hershberger et al., 2021 [49]	hepatocellular carcinoma	unstimulated whole saliva collected using the DNA Genotek OMNIgene ORAL OM-505 after a standard mouth rinse	NR	GC-TOF-MS	down: acetophenone, octadecanol, lauric acid, 3-hydroxybutyric acid, threonic acid, glycerol-alpha-phosphate, butylamine, alphatocopherol
Bel’skaya et al., 2021 [50]	lung cancer	unstimulated whole saliva 5 mL collected by spitting into sterile polypropylene tubes; collection of saliva samples was carried out on an empty stomach after rinsing the mouth with water at 8:00–10:00 a.m.	centrifuged at 10,000× *g* for 10 min, biochemical analysis immediately performed without storage and freezing	StatFax 3300 semi-automatic biochemical analyser	diene conjugates, uric acid (depending on the smoking history and the severity of COPD)
Jiang et al., 2021 [51]	lung cancer	unstimulated whole saliva collected in SalivaGetinTM device by passive drooling at 8:30–10:30 a.m.	centrifuged at 8000× *g* for 10 min at 4 °C, then the resulting supernatant mixed with ACN and ultrapure water; the mixture vortexed for 10 min and centrifuged at 8000× *g* for 10 min at 4 °C once again and stored in the refrigerator at −80 °C until analysis	ultralow noise TELDI-MS	ELC: up: adenine, guanine, cytosine, uracil, creatinine, γ-aminobutyric acid, allysine, gentisic acid, imidazolepropionic acid, ketoleucine, *N*-acetylhistidine, *N*-acetylproline, 3-hydroxyanthranilic acid, and pyroglutamic acid; down: glycyl-phenylalanine, *N*-acetyltaurine, acetyl-L-glutamic acid, phenylgloxylic acid, proline, valine, arginine, serine, and xanthine
Takamori et al., 2022 [52]	lung cancer	unstimulated whole saliva 4–5 mL collected into 50-cc Falcon tubes kept in paper cups filled with crushed ice for 5–15 min after rinsing the mouth with water	centrifuged and immediately stored at −80 °C	CE-TOF-MS	up: diethanolamine; down: tryptophan (*p*-value < 0.05), choline, thymine, cytosine, phenylalanine, leucine, isoleucine, lysine, tyrosine
Zhang et al., 2021 [53]	papillary thyroid cancer	unstimulated whole saliva 1.5 mL collected with Salivette^®^ polyester swabs held in mouth for 5 min after rinsing the mouth with water at 8:30–10:30 a.m.	centrifuged at 3000 rpm for 3 min and at 4 °C, stored at −35 °C until analysis	UPLC-HRMS	Down: L-valine and L-alanine (*p*-value < 0.001), L-phenylalanine, L-proline, L-leucine, L-tryptophan, L-threonine and L-glycine (*p*-value < 0.01), L-methionine, and L-isoleucine (*p*-value < 0.05)
García-Villaescusa et al., 2018 [54]	glioblastoma	unstimulated whole saliva collected in a wide-necked sterile container (“draining method”) in the morning, then transferred with a pipette to a sterile 1.5 mL Eppendorf tube	immediately stored at −80 °C until analysis	NMR spectroscopy	up: propionate and acetate; down: leucine, valine, isoleucine, alanine, ethanolamine, and sucrose

NR, not reported; HS-SPME, headspace solid-phase microextraction; GC-MS, gas chromatography–mass spectrometry; CE-TOF-MS, capillary electrophoresis time-of-flight mass spectrometry; LC-QQQMS, liquid chromatography coupled with triple quadrupole mass spectrometry; HPAEC-PAD, high-performance anion-exchange chromatography with pulsed amperometric detection; UPLC-ESI-MS, ultra-performance liquid chromatograph electrospray ionisation–mass spectrometry; MS, mass spectrometry; LC-Q-TOF-MS, liquid chromatography coupled with quadrupole time-of-flight mass spectrometry; HILIC-UPLC-MS, ultra-performance liquid chromatography–mass spectrometry in hydrophilic interaction chromatography mode; RP-UPLC-ESI-MS, reversed-phase ultra-performance liquid chromatography electrospray ionisation–mass spectrometry; HPLC-MS, high performance liquid chromatography–mass spectrometry; SERS, surface enhanced Raman scattering; GC-TOF-MS, gas chromatography time-of-flight mass spectrometry; TELDI-MS, tip-enhanced laser desorption/ionization–mass spectrometry; UPLC-HRMS, ultra-high performance liquid chromatography–high resolution mass spectrometry; NMR spectroscopy, nuclear magnetic resonance spectroscopy; BC, breast cancer; LC, lung cancer; PC, pancreatic cancer; EGC, early gastric cancer; AGC, advanced gastric cancer; GC, gastric cancer; CRC, colorectal cancer; COPD, chronic obstructive pulmonary disease; ELC, early lung cancer.

**Table 4:** Determined predictive parameters for most discriminant metabolites from included studies.

Study	Oncological Diagnosis	Most Discriminant Metabolites	AUC	−95% CI	+95% CI	Sensitivity [%]	Specificity [%]
Murata et al., 2019 [39]	breast cancer	Spermine	0.766	0.671	0.840	-	-
		Spermine + ribulose-5-phosphate	0.790	0.699	0.859	-	-
Ragusa et al., 2021 [40]	breast cancer	Glucosamine + mannose	0.981	0.911	1.000	-	-
		Glucosamine + mannose + galactose	0.980	0.934	1.000	-	-
		Glucosamine + mannose + galactose + fucose	0.986	0.957	1.000	-	-
		Glucosamine + mannose + galactose + fucose + galactose + galactosamine	0.997	0.989	1.000	-	-
	lung cancer	Mannose + fucose	0.869	0.781	0.943	-	-
		Mannose + fucose + galactose	0.917	0.835	0.982	-	-
		Mannose + fucose + galactose + galactosamine + glucosamine	0.918	0.829	0.976	-	-
Sugimoto et al., 2010 [41]	breast cancer	C_7_H_8_O_3_S + lysine + C_30_H_62_N_19_O_2_S_3_ + threonine + “leucine + isoleucine” + putrescine + C_4_H_12_N_5_ + glutamic acid + tyrosine + piperideine + valine + glycine + C_30_H_55_N_27_O_3_S	0.973	-	-	-	-
	pancreatic cancer	Phenylalanine + tryptophan + ethanolamine + carnitine + C_7_H_12_N_2_O_3_	0.993	-	-	-	-
Takayama et al., 2016 [42]	breast cancer	Spermine	0.744	0.666	0.823	68.9	74.4
		Acetylputrescine	0.704	0.624	0.784	60.7	53.5
		Cadaverine	0.693	0.627	0.758	65.6	67.4
		Putrescine	0.688	0.608	0.769	62.3	51.2
		*N*^1^-acetylspermidine	0.678	0.596	0.760	63.9	53.5
Xavier Assad et al., 2020 [43]	breast cancer	PG 14:2	0.733	0.596	0.870	65.22	77.14
		PI 38:7	0.661	0.513	0.809	60.87	71.43
		PS 28:0	0.627	0.464	0.790	47.83	88.57
Zhong et al., 2016 [44]	breast cancer	Monoacylglycerol (0:0/14:0/0:0)	0.929	0.844	1.000	92.6	91.7
		Lysophosphatidylcholine (22:6)	0.920	0.839	1.000	81.5	91.7
		Lysophosphatidylcholine (18:1)	0.920	0.836	1.000	77.8	100.0
		Phytosphingosine	0.879	0.777	0.981	80.8	92.6
		Lysophosphatidylcholine (18:2)	0.868	0.758	0.977	84.6	92.6
		Histidine	0.847	0.736	0.958	96.3	62.5
		Lysophosphatidylethanolamine (18:2/0:0)	0.821	0.706	0.902	92.6	62.5
		*N*-Acetylneuraminic acid	0.795	0.669	0.921	92.6	58.3
		Phosphatidylethanolamine (22:0/20:4)	0.762	0.630	0.894	70.4	75.0
		Phosphatidylcholine (18:1/16:0)	0.750	0.612	0.885	59.3	91.7
Asai et al., 2018 [45]	pancreatic cancer	Alanine + *N*^1^-acetylspermidine + 2-oxobutyrate + 2-hydroxybutyrate	0.887	0.784	0.944	-	-
Chen et al., 2018 [46]	gastric cancer	Taurine + glycine + glutamine + ethanolamine + histidine + alanine + glutamic acid + hydroxylysine + proline + tyrosine	0.900	-	-	-	-
Bel’skaya et al., 2020 [47]	gastric cancer	Acetaldehyde + acetone + methanol + 2-propanol + ethanol	0.839	-	-	-	-
	colorectal cancer		0.857	-	-	-	-
Kuwabara et al., 2022 [48]	colorectal cancer	4-Methyl-2-oxopentanoate + *N*-acetylputrescine + isoleucine + malate	0.840	0.796	0.883	-	-
		*N*^1^*N*^8^-Diacetylspermidine	0.764	0.718	0.809	-	-
		*N*^8^-Acetylspermidine	0.745	0.699	0.790	-	-
		*N*^1^-Acetylspermine	0.727	0.675	0.780	-	-
		*N*^1^*N*^2^-Diacetylspermine	0.684	0.633	0.735	-	-
		*N*^1^-Acetylspermidine	0.667	0.615	0.725	-	-
Hershberger et al., 2021 [49]	hepatocellular carcinoma	Octadecanol + acetophenone + 1-monopalmitin + 1-monostearin	-	-	-	87.9	95.4
		Octadecanol + 1-monopalmatin + 1-monostearin + 4-hydroxybutyric acid	-	-	-	87.9	93.5
Jiang et al., 2021 [51]	lung cancer	*N*-Acetyltaurine	0.990	-	-	-	-
		Xanthine	0.938	-	-	-	-
		*N*-Acetyl-L-glutamic acid	0.927	-	-	-	-
		Glycyl-Phenylalanine	0.914	-	-	-	-
		Gentisic acid	0.905	-	-	-	-
		Cytosine	0.849	-	-	-	-
		Serine	0.847	-	-	-	-
		Imidazolepropionic acid	0.847	-	-	-	-
		Adenine	0.845	-	-	-	-
		Ketoleucine	0.817	-	-	-	-
Takamori et al., 2022 [52]	lung cancer	Tryptophan	0.663	-	-	-	-
		Phenylalanine	0.634	-	-	-	-
		Choline	0.632	-	-	-	-
		Leucine	0.621	-	-	-	-
		Isoleucine	0.620	-	-	-	-
		Lysine	0.620	-	-	-	-
Zhang et al., 2021 [53]	papillary thyroid cancer	Alanine + valine + proline + phenylalanine	0.936	0.894	0.977	91.2	85.2
		Valine	0.833	0.758	0.907	80.3	78.4
		Alanine	0.814	0.736	0.891	72.1	76.5
		Threonine	0.755	0.663	0.848	63.9	92.2
		Proline	0.754	0.665	0.843	50.8	92.2
		Phenylalanine	0.749	0.658	0.839	98.4	43.1

AUC, area under curve; CI, confidence interval; -, not reported.

## Data Availability

Data are available on request from the corresponding author. The data are not publicly available due to this is a systematic review (not an original article), so the database is in Excel and contains the data already displayed in most Tables in our manuscript.
